# Graphene Quantum Dots in Neurodegeneration: Protein Aggregation and the Microglial State as Therapeutic Targets

**DOI:** 10.3390/nano16181158

**Published:** 2026-09-15

**Authors:** Hanna Jeong, Yuri Kang, Seoyu Jeong, Je Min Yoo, Yong Seok Choi, Sang Ho Kwon, Heehong Hwang, Minyeop Nahm, Donghoon Kim

**Affiliations:** 1 Department of Translational Biomedical Sciences, Graduate School of Dong-A University, Busan 49201, Republic of Korea; hanna7680@naver.com (H.J.); kyuri0609@naver.com (Y.K.); jseoyuu@gmail.com (S.J.); 2 Department of Pharmacology, College of Medicine, Dong-A University, Busan 49201, Republic of Korea; 3 Chaperone Ventures LLC, Los Angeles, CA 90010, USA; jeminyoo@chaperone.ventures; 4 SmartinBio Inc., Cheongju 28160, Republic of Korea; 5 Graphene Research Center, Advanced Institute of Convergence Technology, Suwon 16229, Republic of Korea; cyscell@gmail.com; 6 Biochemistry, Cellular and Molecular Biology Graduate Program, Johns Hopkins School of Medicine, Baltimore, MD 21205, USA; kwonsangho1107@gmail.com; 7 Department of Neuroscience, Interdisciplinary Program in Neuroscience, Seoul National University, Seoul 08826, Republic of Korea; hhwang11@gmail.com; 8 Dementia Research Group, Korea Brain Research Institute, Daegu 41062, Republic of Korea

**Keywords:** graphene quantum dots, neurodegenerative disease, protein aggregation, microglia, neuroinflammation

## Abstract

Graphene quantum dots (GQDs) constitute the zero-dimensional family of carbon nanomaterials, combining efficient penetration of the blood–brain barrier (BBB) with precisely controllable surface chemistry, and have drawn attention as theragnostic platforms that carry both diagnosis and therapy in a single particle. Although various neurodegenerative diseases each arise from a different causal protein, they converge on shared pathology: aberrant accumulation of neuronal proteins and chronic neuroinflammation driven by pathological activation of glial cells such as microglia and astrocytes. GQDs modulate aggregation through monomer capture that delays nucleation, the blockade of growing fibril tips, and the penetration and disassembly of mature fibrils, all of which have been verified against various protein aggregation models. Beyond aggregation control, heteroatom doping and surface functionalization design allow GQDs to scavenge reactive oxygen species (ROS) and adjust autophagic pathways, attenuating markers of pathological microglial activation. Tunable emission simultaneously enables imaging of pathological burden and high-sensitivity detection of fluid biomarkers, supporting integration of therapeutic and diagnostic functions. GQD bioactivity, however, is inherently double-edged: depending on composition and concentration, the same materials can trigger mitochondrial oxidative stress and ferroptosis rather than exerting antioxidant action, as observed in microglia and macrophages. Therefore, key challenges remain separating anti-inflammatory efficacy from toxicity through precise surface functionalization, ensuring batch-to-batch reproducibility, establishing standardized physicochemical reporting, and quantitatively delineating the therapeutic window.

## 1. Introduction

### 1.1. Biomedical Applications of Nanomaterials

Drug therapy remains the cornerstone of disease management in modern medicine. Historically, the medical field has relied on natural products as therapeutics. Once chemical synthesis and molecular biology matured, these products were refined and reengineered to support the continuous development of approved pharmaceuticals [1,2]. Natural compounds and their derivatives still account for a substantial fraction of newly approved drugs, maintaining this pipeline as a central pillar of drug discovery [3]. Concurrently, the shift toward precision medicine, in which treatment is tailored to the individual patient, has positioned nanotechnology as a key enabler for these advancements [4]. Nanomaterials are commonly defined by dimensions ranging from 1 to 100 nm, and at this length scale their surface area per unit mass rises dramatically while distinctive physical and chemical behaviors that are absent in bulk form emerge [5,6]. Biomedical utilization of these properties has unfolded along three directions. The first is targeted delivery, in which a drug is carried to specific cells to boost efficacy while limiting systemic exposure [7,8]; the second is high-sensitivity sensing and contrast imaging for diagnostics [9]; and the third is the use of biocompatible scaffolds that support cell growth and tissue regeneration [10].

Among the many classes of nanomaterials, carbon-based materials have advanced most rapidly in biomedical research. Depending on carbon bonding configurations and the dimensionality of the resulting structure, they are classified into zero-dimensional fullerenes and carbon quantum dots, one-dimensional carbon nanotubes, and two-dimensional graphene and graphene oxide [11,12]. They share common features such as robust structural integrity, considerable chemical flexibility achieved through surface modification, and redox activity originating from their pi-electron systems, attributes that together make them attractive platforms for biomedical applications and have prompted efforts to apply them to the diagnosis and treatment of neurodegenerative diseases [13,14,15]. Biocompatibility, however, varies substantially with the specific material and its surface chemistry, and thus assuming uniformly low toxicity across the entire family would be unjustified [16,17].

Beyond biocompatibility concerns, a fundamental challenge also hinders nanomedicine translation: successful preclinical outcomes do not reliably convert into clinical efficacy. A principal reason is that the biological identity a nanoparticle acquires in circulation differs from the one it was designed with, so in vivo behavior cannot be predicted from physicochemical characterization alone [18,19]. Recent proposals for a staged translational framework, sequentially verifying physicochemical characterization, target-tissue exposure, formulation scalability, and regulatory readiness, have begun to reset the evaluation standards for nanomaterial research [20]. The GQDs examined in this review must be judged within this same framework. In this review, the term GQD is defined specifically as crystalline, *sp*^2^-bonded graphitic nanoparticles. These particles must have lateral dimensions of approximately 20 nm or smaller and maintain a platelet-like graphitic lattice.

### 1.2. Discovery of GQDs and Their Biomedical Applications

Progress in carbon nanoscience has closely paralleled the discovery of new carbon allotropes. Laser vaporization first revealed C60 fullerene in 1985 [21], and the 1990 breakthrough that enabled large-scale production through the arc-discharge method initiated the modern era of carbon nanomaterials research [22]. Carbon nanotubes (CNTs) followed in 1991 [23], and their applications rapidly expanded into drug delivery and imaging [24]. In 2004, Novoselov and Geim isolated single-layer graphene by mechanical exfoliation and characterized its electrical behavior [25]. Graphene consists of carbon atoms arranged in a hexagonal honeycomb lattice forming a two-dimensional plane. This sequence of discoveries laid the groundwork for graphene oxide (GO)-based drug delivery systems and, ultimately, for GQDs, shaping the trajectory of contemporary carbon nanotechnology [26].

Reducing the lateral dimensions of graphene flakes to below approximately 20 nm converts them into GQDs, carbon nanostructures approaching zero dimensionality. At such sizes, quantum confinement emerges, opening an energy band gap that pristine graphene lacks and endowing the dots with the ability to absorb light and emit characteristic fluorescence [27,28]. The emission is now recognized as a complex phenomenon in which quantum confinement cooperates with edge states, surface functional groups, and defect levels, and it manifests as tunable features such as excitation-wavelength dependence and pH sensitivity [29,30,31,32]. Exploiting this luminescence has offered a route to environmentally benign QD-LEDs and flexible transparent displays, so GQDs garnered early attention as next-generation display materials [33].

The earliest biomedical use of GQDs utilized these optical properties for high-resolution bioimaging [34,35]. Organic fluorescent dyes had suffered from pronounced photobleaching, their emission fading over time, while the cadmium-based quantum dots developed to overcome that limitation raised toxicity concerns due to the leakage of heavy-metal ions, restricting their in vivo use. Poor renal clearance compounded the problem, allowing such particles to persist in the body for extended periods [36]. GQDs, by contrast, combine strong photostability with comparatively minimal cytotoxicity, offering a favorable profile for biological use [37,38]. Beyond imaging and sensing, recent work has investigated them as drug carriers, as photothermal and photodynamic therapeutics, and as agents that modulate the aggregation of proteins linked to neurodegenerative diseases [39,40]; the aggregation-modulating activity, in particular, became a central research theme after its first demonstration in vivo [40].

In the literature, GQDs are frequently conflated with carbon dots, graphene oxide quantum dots, and carbon nano-onions. All of these are fluorescent carbon nanoparticles, but GQDs specifically denote a structure in which the *sp*^2^-bonded graphitic lattice is preserved as a crystalline platelet while only the lateral size is reduced; this distinguishes them structurally from carbon dots, which possess an amorphous carbon core [41]. The distinction matters because the degree of π-electron delocalization, redox activity, and stacking interactions with protein surfaces are directly governed by these structural features. In practice, however, synthesis routes and nomenclature are applied inconsistently, so materials carrying the same name often display different properties [42]; comparing results across studies therefore demands that the reported physicochemical data be carefully evaluated on an individual basis. This stems from the fact that synthesis conditions can induce significant structural and surface heterogeneity in graphite-based materials [43,44].

To navigate the conceptual inconsistencies prevalent in the current literature, this review prioritizes terminological precision. We define pathological microglial activation as a chronic phenotypic shift characterized by persistent pro-inflammatory cytokine release, morphological alterations, and diminished homeostatic surveillance. We move beyond the outdated M1/M2 dichotomy, which single-cell analysis has shown to diverge from actual transcriptional states. Autophagy modulation is strictly partitioned into induction (increased autophagosome formation) and flux restoration (reestablished lysosomal degradation). Furthermore, we distinguish fibril disassembly, requiring direct structural evidence via TEM, AFM, or NMR, from mere inhibition of elongation, as Thioflavin T fluorescence reductions alone only reflect decreased β-sheet content. Finally, all conclusions are tied to specific material preparations rather than being generalized across the GQD class.

### 1.3. Shared Pathological Mechanisms of Neurodegenerative Diseases

Longer life expectancies driven by population aging are causing a significant rise in the prevalence of neurodegenerative diseases. The global number of individuals with dementia is projected to increase from approximately 57.4 million in 2019 to 152.8 million by 2050, making it a critical global public health challenge [45]. Similarly, the overall burden of neurological diseases continues to rise steadily [46]. AD, PD, ALS, and HD, the primary focus of this review, are characterized by progressive neuronal loss. This neurodegeneration is primarily driven by the misfolding and pathogenic aggregation of neuronal proteins such as Aβ, tau, α-synuclein, TDP-43, and huntingtin [47,48]. Such aggregates are known to spread pathology from cell to cell [49].

Pathological aggregation of neuronal proteins acts as a key trigger of neuroinflammation [50]. Over the past few decades, research focus has expanded from neurons alone to the broader neuroinflammatory and brain microenvironment [51]. Consequently, microglia, the resident macrophage-like cells of the central nervous system, have emerged as critical mediators of disease progression [52,53]. In their homeostatic state, microglia maintain brain integrity by trimming synapses and clearing debris [54], but as aggregates accumulate, they shift into disease-associated microglia (DAM) states [55,56,57]. DAM cells secrete inflammatory cytokines, excessively eliminate healthy synapses via the complement pathway [58,59,60], and induce a neurotoxic reactive astrocyte state, thereby accelerating neuronal death [61,62].

Microglia were long divided dichotomously into pro-inflammatory M1 and tissue-repair M2 phenotypes, but single-cell transcriptomics has shown that they do not exist as two fixed states; instead, they shift along a continuous spectrum as disease and aging progress [63,64]. A state-based nomenclature has accordingly been proposed to replace the M1/M2 dichotomy [52], and this review adopts that state-based perspective. Modulating microglial activity toward an anti-inflammatory direction has therefore emerged as a promising therapeutic strategy for multiple neurodegenerative diseases, with a range of small-molecule and antibody-based approaches under investigation [65,66,67].

### 1.4. Limitations of Current Therapeutic Strategies

The history of drug development for neurodegenerative diseases has been limited by single-target thinking. In AD, although monoclonal antibodies targeting Aβ have been approved and have demonstrated reductions in pathological markers and a slowing of cognitive decline, their clinical impact remains modest due to limited effect sizes and safety concerns, such as amyloid-related imaging abnormalities (ARIA), alongside the burden of intravenous administration [65,66]. In PD, dopamine replacement relieves symptoms but does not modify the course of the disease, and antibodies directed at α-synuclein have failed to demonstrate significant clinical efficacy [68,69]. Three shared factors account for these shortcomings. First, the large molecular weight of antibodies yields very low blood–brain barrier (BBB) transport efficiency, making it hard to reach effective concentrations within the brain. Second, because they primarily engage extracellular aggregates, they have little effect on intracellular aggregates or on mature fibrils that have already formed. Third, they fail to address both pathological axes, protein aggregation and neuroinflammation, simultaneously. What is needed, then, is a material that crosses the BBB, enters cells, and couples aggregate disassembly with immune modulation; the small size and tunable surface chemistry of GQDs make them highly attractive for these reasons [70]. This review examines the role of GQDs in modulating protein aggregation and neuroinflammatory cascades. We investigate the competing effects of GQD bioactivity, specifically the balance between beneficial redox/autophagic modulation and potential cytotoxicity or ferroptosis. This insight is key to designing safe agents for neurodegenerative diseases. Along these lines, this review examines the fabrication and biological behavior of GQDs, therapeutic strategies addressing both pathological axes alongside diagnostic integration and safety concerns, and the future outlook.

Original research and review articles on the synthesis, biological behavior, and neurotherapeutic applications of GQDs were prioritized (detailed criteria are available in the Supplementary Materials). 

## 2. Structure, Synthesis, and Biological Behavior of GQDs

### 2.1. Structure, Properties, and Synthetic Routes

**Figure 1:**
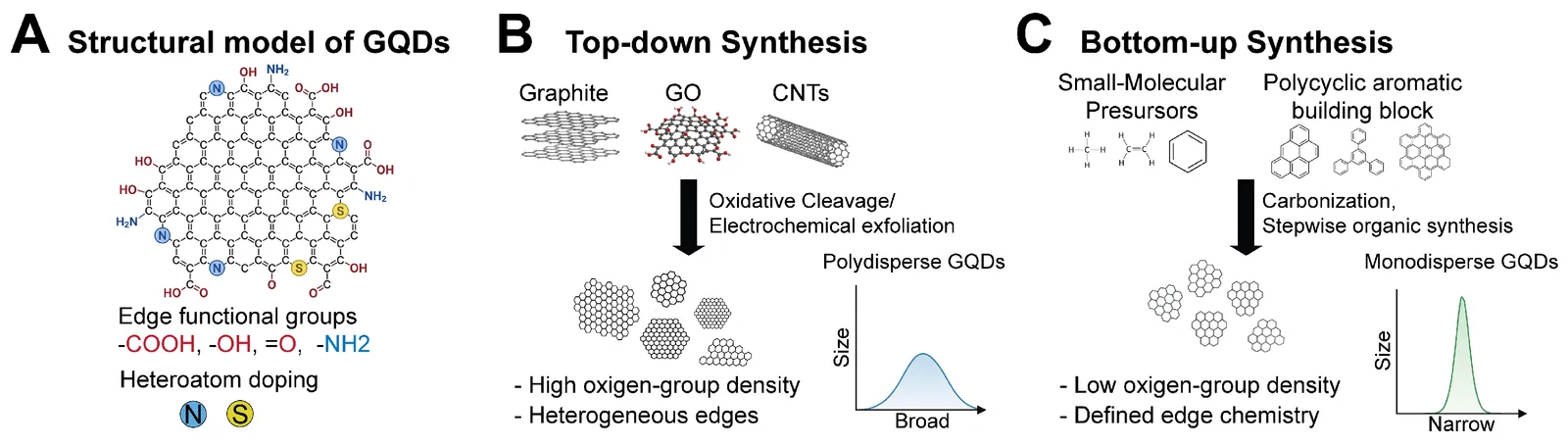
Structure and synthetic routes of GQDs. (**A**) Structural model showing the crystalline platelet of the *sp*^2^ graphitic lattice with edge functional groups (e.g., –COOH, –OH, =O, and –NH_2_) and heteroatom doping sites (e.g., Nitrogen and Sulfur). (**B**) Top-down route: preparation from graphite, graphene oxide, and carbon nanotubes by oxidative cleavage and electrochemical exfoliation. (**C**) Bottom-up route: carbonization of small-molecule precursors and stepwise organic synthesis. Differences in surface chemistry and size distribution introduced by each route are indicated.

As zero-dimensional (0D) carbon materials derived from graphene sheets, GQDs display distinctive optical and physical characteristics that arise from quantum confinement and edge effects at the nanometer scale. These characteristics can be engineered by varying the density of oxygen-containing groups, doping with heteroatoms such as nitrogen, sulfur, or boron, and tailoring the edge structure (Figure 1A) [30,31,41].

The photoluminescence of GQDs arises from a complex interplay of multiple mechanisms rather than a single origin. While quantum confinement within *sp*^2^ domains is a primary driver, emission is often modulated by competing pathways, such as defects, edge states, surface functional groups, and molecular fluorophores. This complexity is compounded by heteroatom doping, where emission is dictated by specific bonding configurations rather than total dopant concentration. Because common optical signatures (e.g., pH sensitivity) are not unique to quantum confinement, distinguishing these mechanisms requires rigorous decoupling through time-resolved spectroscopy and stringent purification. The inconsistent application of these controls in biomedical studies, however, remains a significant barrier to comparing results across the literature.

Synthetic strategies for GQDs fall into two broad categories (Figure 1B,C). In top-down approaches, large carbon precursors such as graphite, graphene oxide, carbon fibers, and carbon nanotubes are fragmented into nanometer-sized particles via acid-assisted sonication, solvothermal treatment, electrochemical exfoliation, or oxidative cleavage [29,33]. These methods are comparatively amenable to mass production and introduce oxygen-rich groups, but achieving precise size control remains challenging, and the chemical composition of the edges is difficult to manage [71]. In contrast, bottom-up approaches synthesize GQDs from small precursors, such as citric acid, glucose, and amino acids, through carbonization, pyrolysis, or stepwise organic synthesis [72,73]. These routes offer better control over size and composition and favor heteroatom doping, although the processes are complex and scaling up production is challenging [33,41]. The salient features of the principal methods are summarized in Table 1.

Synthesis conditions, such as temperature, pH, precursor concentration, reaction time, and dopant introduction, determine both the size and surface properties of the GQDs, which in turn exert a direct effect on biological behavior. For instance, the composition of oxygen-containing groups has been shown to directly alter the ability to inhibit protein aggregation [74], while the type of surface functionality can modulate cytotoxicity significantly at identical concentrations [75]. This tunability is an advantage when designing therapeutic materials but a weakness regarding batch-to-batch reproducibility. Across the carbon-nanoparticle field, investigators have observed that subtle differences in synthesis conditions lead to property variation that complicates cross-study comparisons, underscoring the need for standardized minimum reporting items [42]. To enable meaningful inter-study comparisons, a standardized characterization framework is essential. Primary particle size should be determined via HR-TEM and AFM rather than inferred from DLS, which is better suited for evaluating hydrodynamic behavior in biological media. Furthermore, zeta potential data must specify the measurement pH and ionic strength, alongside comprehensive reporting on surface chemistry, Raman D/G ratios, quantum yield, and residual contaminants such as metals and endotoxins.

### 2.2. Behavior in Biological Environments

Existing studies consistently indicate that the biological behavior of GQDs is dictated by their physicochemical properties. Size is particularly influential, governing BBB crossing and cellular uptake in drug delivery for neurodegenerative disease [13,70], while renal excretion is also strongly dependent on it. Studies on cadmium-based quantum dots quantified that hydrodynamic diameters at or below 5.5 nm undergo rapid urinary elimination, whereas the adsorption of serum proteins that increases the diameter by more than 15 nm blocks excretion [36]. The 5.5 nm renal clearance threshold observed in cadmium-based quantum dots is specific to rigid, spherical structures and has not been replicated in GQDs. This discrepancy arises because GQDs possess fundamentally different characteristics such as platelet geometry, deformability, and oxygen-rich surfaces that are filtered differently than conventional quantum dots. Consequently, the 5.5 nm value should be viewed merely as an order-of-magnitude reference rather than a direct threshold applicable to GQDs.

Surface charge and functional groups determine in vivo protein interactions, biological membrane binding, and target biomolecule recognition. When nanoparticles are exposed to plasma, a protein corona forms, masking the intended surface functionality [18], with its composition varying according to particle size and surface chemistry [19,76]. Furthermore, the redox activity stemming from the π-electron sharing of the hexagonal carbon lattice and in oxygen-containing groups allows GQDs to participate directly in electron-transfer reactions and scavenge reactive oxygen species (ROS) [77,78]. From a pharmacokinetic standpoint, particle size and functional groups directly define the absorption, distribution, metabolism, and excretion (ADME) profile; animal studies have indeed reported that biodistribution and organ retention following intravenous and intraperitoneal administration exhibit material-dependent variations [16,79].

The colloidal and chemical stability of GQDs in biological media remains an overlooked aspect of their characterization. As these particles transit through varying pH and redox environments, they are prone to structural changes ranging from electrostatic-driven aggregation to the actual degradation of the graphitic core. Such transformations can induce fluorescence quenching, meaning a decline in signal cannot be blindly equated to clearance without independent verification. Despite this, most reviewed studies fail to provide the critical metrics such as hydrodynamic diameter, zeta potential, and XPS O/C ratios required to track these changes accurately.

Building on these properties, GQDs deliver several biomedical functionalities simultaneously. Tunable emission, reduced toxicity, and robust photostability enable precise bioimaging and image-guided therapy [35,37,80]. High specific surface area and abundant functional groups support efficient drug and gene delivery [26,81], and BBB permeability confers value as a theranostic platform for central nervous system diseases [38,82]. Their environment-responsive properties also facilitate high-sensitivity sensing of a variety of biological markers [83,84,85]. The details of these diagnostic uses are discussed below, in the context of diagnostic imaging applications.

### 2.3. Blood–Brain Barrier Transport and Pharmacokinetics

The primary determinant for applying GQDs in the central nervous system is BBB transport, and the underlying mechanism is set by the combination of size, surface charge, and hydrophobicity. Particles with lateral dimensions below 10 nm and optimized hydrophobicity can traverse the barrier by passive transcellular diffusion, and ligand-functionalized versions exploit receptor-mediated transcytosis [70]. Even with targeting ligands such as transferrin, the protein corona that forms in vivo can mask ligand-receptor recognition and reduce transport efficiency [76], so surface design must be evaluated considering corona formation. It is a mistake to rely solely on size to predict GQDs’ behavior in vivo. The reality of transport is much more dynamic, involving changes in hydrodynamic diameter, surface charge shifts, the formation of protein coronas, and particle aggregation, all of which interact with the administration route and disease-altered biological barriers. To illustrate, a 10 nm cationic particle in the bloodstream can act more like a 30 nm neutral particle. Ultimately, evaluating penetration through the BBB requires a comprehensive ‘size-shape-surface’ framework. Once in circulation, the biological identity of GQDs is largely dictated by protein corona formation. The resulting expansion in hydrodynamic diameter quickly surpasses the physical aperture of intact blood–brain barrier tight junctions (~1–2 nm), ruling out paracellular diffusion while sterically obstructing receptor-mediated transcytosis. Concurrently, opsonization shifts uptake toward scavenger pathways and accelerates reticuloendothelial clearance, markedly shortening systemic half-life. This adsorbed protein layer also passivates the graphitic lattice, sterically hindering the π-π and electrostatic contacts required to bind and destabilize pathogenic protein aggregates. Paradoxically, while surface passivation suppresses acute membrane disruption and reactive oxygen species generation, it promotes hepatic and splenic sequestration, shifting the primary safety concern from acute cytotoxicity to long-term tissue accumulation.

Intranasal delivery through the olfactory and trigeminal routes has been proposed as an alternative to systemic administration, bypassing both first-pass hepatic metabolism and the BBB [86,87]. Applying the excretion threshold quantified above, particles that exceed it accumulate preferentially in the liver and spleen instead of being eliminated in urine [36]; the corresponding in vivo safety data are examined below, together with the therapeutic window. In summary, GQD design requires a balance between the size range favorable for BBB transport and that required for renal excretion, a balance that plays a critical role in determining the breadth of the therapeutic window.

## 3. GQD-Based Strategies for Neurodegenerative Disease Treatment

### 3.1. Direct Modulation of Pathogenic Protein Aggregation

Graphene oxide has been studied as an inhibitor of amyloid seeding, utilizing the large hydrophobic surface and dense oxygen-containing groups characteristic of its two-dimensional sheet structure [74,88]. By capturing monomers and oligomers on its broad planar surface, it acts as an inhibitory surface that arrests fiber growth, and in vitro experiments across several disease models suggest that it can control the early stages of aggregation effectively [89,90].

For clinical translation, however, several structural limitations remain. The extensive hydrophobic surface makes nonspecific plasma-protein adsorption, and hence the corona formation described above, particularly pronounced [18]. Furthermore, dimensionality and surface-charge conditions can shift the material’s role from inhibiting to promoting aggregation [91]. Additionally, the chirality of the surface has been found to steer the aggregation pathway [88,92]. The physical dimensions of the two-dimensional sheets also pose challenges for the precise control of in vivo accumulation and long-term biodistribution [93].

GQDs have emerged as promising alternatives that address these limitations and enable more precise control over proteins. Like graphene oxide, they engage in protein aggregation through π-π stacking and hydrophobic interactions, yet their zero-dimensional form confers a distinct mode of action [88,94]. Owing to their small size, they can cap the growing tips of amyloid fibrils to halt elongation, or penetrate established fibrils and extract peptides to drive disassembly [40,90,95,96]. Their surface can also be functionalized with high versatility, permitting selectivity toward particular disease proteins [97,98,99,100,101].

**Figure 2:**
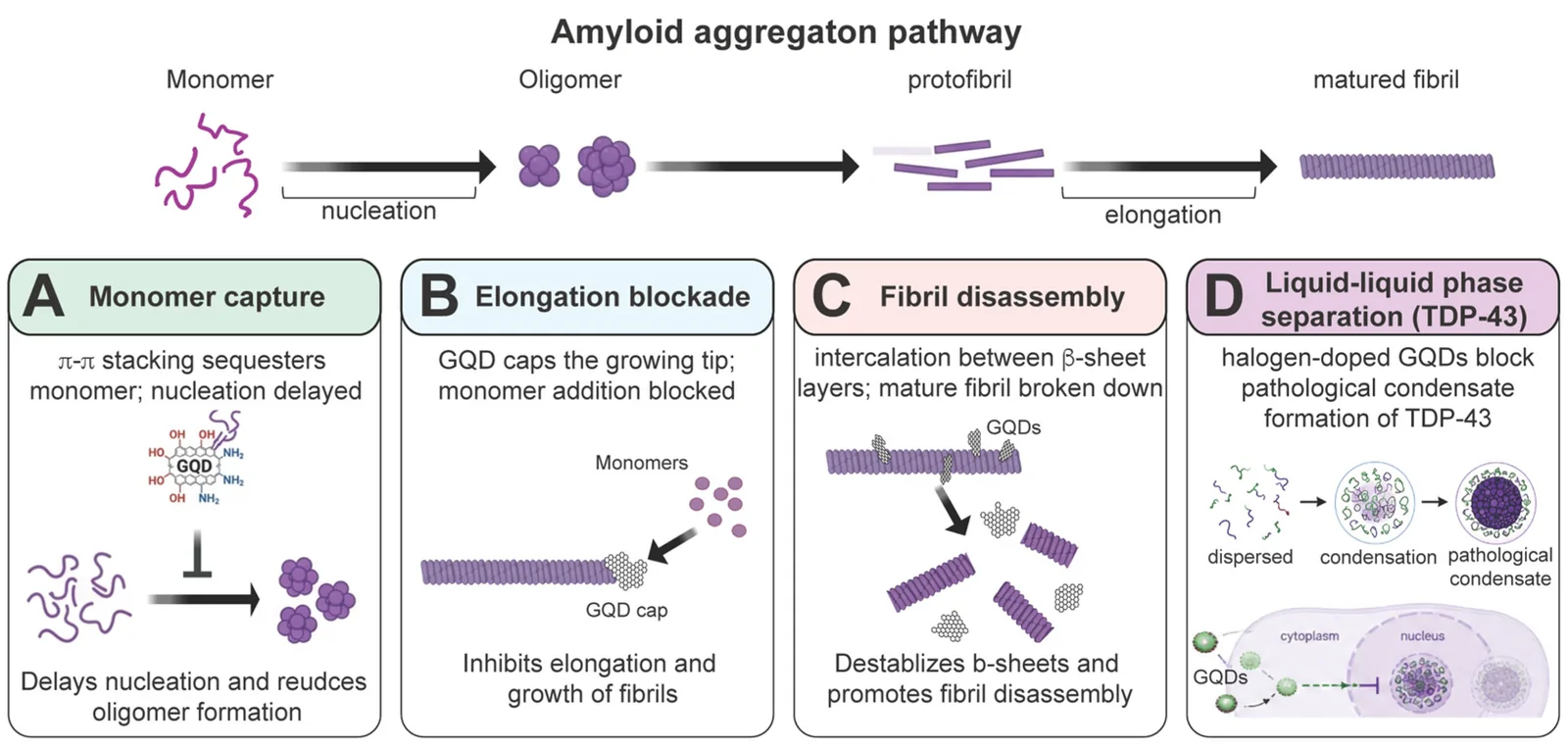
Mechanisms of amyloid aggregation control by GQDs. Four sites of action are mapped onto the aggregation pathway: monomer to oligomer to protofibril to mature fibril. (**A**) Monomer capture by π-π stacking and delayed nucleation, (**B**) blocking of elongation by tip binding, (**C**) fibril disassembly through intercalation between β-sheet layers, and (**D**) acting on the liquid–liquid phase separation step to block pathological condensate formation (specifically for TDP-43).

The mechanisms by which GQDs modulate amyloid aggregation can be grouped into three categories (Figure 2A–D). First, monomer capture via π-π stacking and hydrophobic interactions delays nucleation [74,90]. Second, binding to the growing fiber ends blocks elongation [40]. Third, intercalation between the β-sheet layers of mature fibrils disturbs the hydrogen-bonding network and extracts peptides, inducing dissociation [40]. Beyond these classical mechanisms, recent findings suggest that intervention at the liquid–liquid phase separation (LLPS) stage prevents the formation of pathological condensates altogether; this mechanism, documented for TDP-43, marks a point of action distinct from the classical amyloid pathway [100,102]. The relative contribution of each mechanism depends on the target protein and surface chemistry: the density of oxygen-containing groups governs electrostatic interactions [74], halogen doping modulates nuclear translocation and phase-separation control [100], chiral surface arrangements enable stereoselective binding, as demonstrated for enantiomeric carbon dots [103], and in silico simulations have confirmed that the degree of dimerization inhibition varies with the functional group [94].

Table 2 summarizes the validation status for each target protein. Against α-synuclein, GQDs suppress fibrillization, induce disassembly of mature fibrils, and mitigate neuronal death and synaptic loss in animal models [40]. For Aβ, the intrinsic inhibitory effect of GQDs [90] is synergistically enhanced by conjugates with tramiprosate [98], and nitrogen doping alters anti-amyloid potency [99]. For tau, biomimetic graphene nanoparticles block cell-to-cell transmission [104], and a strategy of loading D-cysteine-functionalized GQDs into extracellular vesicles has been proposed to improve targeting [105]. For TDP-43, halogen-doped GQDs traverse the nuclear membrane to regulate intranuclear aggregation and phase separation [100], and they alleviate stress-granule-mediated cytoplasmic aggregation in motor neurons [102]. Favorable effects were also reported in a Niemann-Pick type C model characterized by lysosomal cholesterol accumulation, demonstrating that the range of action extends beyond classical amyloidogenic diseases [106].

Validation levels are categorized into in silico (computational simulation), in vitro (cell-free assays), cellular (cell lines or primary cultures), and animal (rodent or other model organisms). To date, no clinical data involving human subjects have been reported.

We classify mechanistic claims based on the rigor of their supporting evidence. A mechanism is directly demonstrated if high-resolution structural data (e.g., cryo-EM, ssNMR, or AFM/TEM) resolve specific binding geometries. Mechanisms from ensemble-averaged assays (e.g., ThT fluorescence, turbidity, or CD) are inferred, as they reflect population-level behavior rather than discrete binding sites. Finally, mechanisms based on computational simulations are predicted. Under this framework, tip capping and intercalation are directly demonstrated for α-synuclein but remain inferred for most other targets; similarly, functional-group selectivity is treated as predicted rather than measured.

While preliminary evidence is largely confined to in vitro and rodent studies, there is a conspicuous absence of systematic research exploring the direct interaction between GQDs and huntingtin, the primary driver of HD. Aggregation driven by polyglutamine (polyQ) expansion follows different kinetics from Aβ or α-synuclein, so extrapolating existing results is challenging. Verification in HD and other polyglutamine disorders therefore remains a significant gap for future research [48].

Due to a lack of quantitative thermodynamic data, structure–activity relationships in this field remain largely qualitative. Most existing studies rely on fluorescence quenching, which is problematic as it conflates binding with quenching effects and fails to provide essential enthalpy and entropy information. To achieve quantitative rigor, it is necessary to employ techniques such as ITC, SPR, QCM-D, NMR, and molecular dynamics to accurately determine binding enthalpy, kinetics, and free-energy profiles.

### 3.2. Pathological Microglial Activation and Its Significance as a Therapeutic Target

Building on the shared aggregation-neuroinflammation axis outlined in the Introduction [47,50], the following discussion examines why microglial state modulation has emerged as a therapeutic target.

Three considerations explain the growing interest in microglia-directed strategies. First, microglial activation is a pathological hallmark irrespective of the specific disease, so unlike approaches aimed at a single causal protein, it could in principle apply to multiple disorders [51,52]. Second, the DAM state is not an irreversible endpoint but a reversible transcriptional transition, making state switching by intervention feasible in principle [55,56,57]. Third, because microglia are positioned upstream of neuronal damage by mediating synapse elimination and astrocyte activation [58,59,60,61,62], modulating this node can influence downstream pathology broadly.

However, since microglial states constitute a continuous spectrum [63,64], selectively adjusting a single state is technically challenging. Pathological activity must be relieved without undermining homeostatic functions [54], which calls for interventions precise enough to be guided by state-based markers [107,108]. The difficulty that conventional small-molecule and antibody approaches have had in achieving such selectivity [65,67] is what motivates interest in nanomaterial-based strategies.

### 3.3. Modulation of Microglial States by GQDs

**Figure 3:**
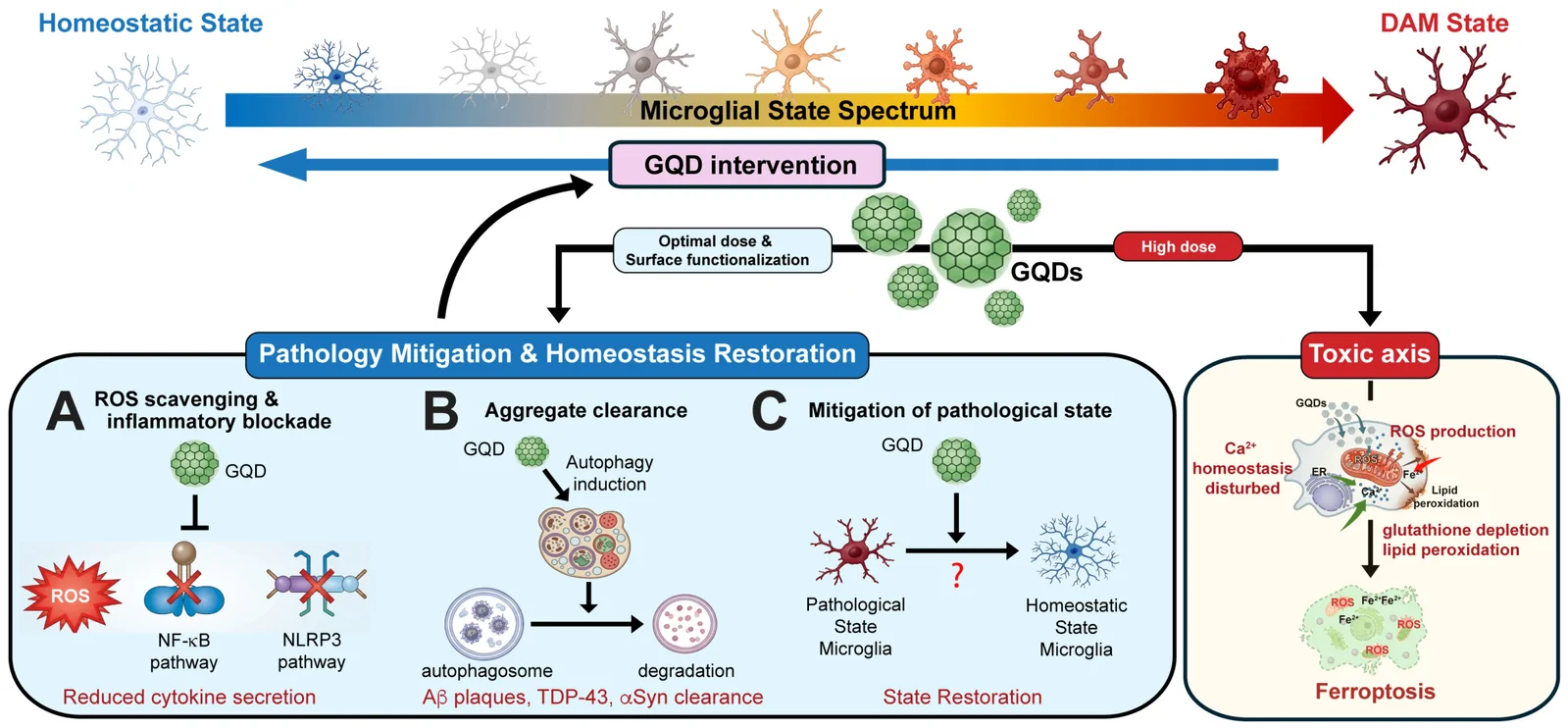
Sites of action in microglial state modulation. The continuous transition between the homeostatic state and the disease-associated microglia (DAM) state is depicted as a spectrum, with three points of action of GQDs. (**A**) Reduced pro-inflammatory cytokine secretion (e.g., IL-1β) through ROS scavenging and blockade of NF-κB and NLRP3 pathways, (**B**) aggregate removal via autophagy induction and reduction in upstream stimuli, and (**C**) potential mitigation of the pathological state and restoration of homeostasis-related gene expression (hypothetical, indicated by ‘?’). The toxicity axis branching from the same pathway into ferroptosis and autophagic cell death is also shown.

Both graphene oxide and GQDs have been investigated for their ability to control pathological microglial states [109,110,111]. Due to their nanometer scale, GQDs readily cross the BBB, and their surface chemistry can be adjusted to design antioxidant and anti-inflammatory properties [40,70]. The reported modes of action fall into three categories (Figure 3A–C).

The first is ROS scavenging together with suppression of downstream inflammatory signaling. GQDs doped with nitrogen or halogen elements neutralize intracellular reactive oxygen species [77,78], acting upstream of the NF-κB and NLRP3 inflammasome pathways whose activation and consequent IL-1β release are established features of these diseases [112,113,114]. A nitrogen-doped construct functionalized with the RGDS peptide exhibited intracellular antioxidant activity even at a concentration as low as 10 μg/mL [77].

The second mechanism involves aggregate removal through induction of autophagy. By activating autophagic pathways within cells [78,115], GQDs promote clearance of pathological material such as TDP-43 aggregates [100], thereby lowering the upstream stimuli that drive DAM conversion. However, since the therapeutic benefit of aggregate clearance and the toxicity of autophagic cell death stem from the same pathway, the intensity of induction must be carefully controlled [75].

The third mechanism involves the modulation of cellular states. In a Niemann-Pick type C model, GQD treatment reduced microglial activation alongside functional rescue [106], indicating that the material acts on pathological microglial states, although DAM-specific markers were not assessed in that study. A recurring limitation in the current literature is that activation has been scored using general inflammatory indicators, such as Iba1 immunoreactivity and cytokine release, without resolving the underlying microglial state transitions at the transcriptional level. This underscores the necessity for future research to utilize microarray and RNA-seq technologies to characterize these transcriptional-level transitions across different neurodegenerative disease models. In an experimental autoimmune encephalomyelitis model, intraperitoneal delivery of GQDs at 10 mg/kg/day led to accumulation in the lymph nodes and the central nervous system cells, effectively attenuating the inflammatory response [109]. Although describing these changes as M1-to-M2 repolarization would conflict with the state-spectrum view presented earlier for the shared pathological mechanisms and for microglial activation, this review frames these transitions as a restoration of homeostatic gene expression, rather than a dichotomous conversion between phenotypes [52].

The same redox activity that mediates ROS scavenging in microglia can, at higher concentrations, drive those cells into ferroptosis through mitochondrial oxidative stress and disturbed calcium homeostasis [116,117]; the general form of this efficacy-toxicity duality is treated below, in the discussion of safety and toxicity. Future work should therefore focus on surface engineering that minimizes toxicity while selectively targeting microglia in pathological states, and on delivery systems that ensure both BBB transport efficiency and long-term safety [20,86,87]. Successfully addressing these challenges could enable microglial state control to transcend single-protein targeting and evolve into an approach that reshapes the immune microenvironment of the brain.

### 3.4. Diagnostic Imaging Applications and Theranostic Integration

The therapeutic use of GQDs cannot be separated from their diagnostic function. Nitrogen-doped dots combine Aβ-binding capacity with fluorescence, enabling the imaging of protein aggregates. For instance, nitrogen-doped GQDs with a particle size of 7.4 nm and a quantum yield of 57.3% retained high photostability while keeping cytotoxicity below 10% even at 250 μg/mL, addressing the limitations of organic-dye-based Aβ imaging probes [118]. Enantioselective targeting of amyloid has been visualized in vivo with chiral non-carbon probes [119], suggesting that chiral surface design could likewise sharpen imaging contrast for GQDs.

Electrochemical and fluorescence-based GQD sensors can detect biofluid biomarkers, including α-synuclein, phosphorylated tau, and neurofilament light chain, at low concentrations, supporting early diagnosis and the monitoring of therapeutic response [83,84,85]. When such diagnostic capacity is integrated into the same nanostructure that performs aggregate disassembly and microglial modulation, the dots become a theranostic platform capable of verifying target delivery and tracking treatment efficacy following a single administration [80,82]. This approach could effectively mitigate the clinical burden of antibody therapies, which currently necessitate separate PET imaging and repeated MRI scans. Two primary challenges remain: first, the therapeutic window discussed below has not been quantified [75,79,120]. Second, the limited tissue-penetration depth of fluorescence requires shifting emission into the near-infrared region for clinical application. While recent developments, such as N-B-GQDs [121], have demonstrated the immense potential of this approach through successful NIR-I/II imaging and cancer therapy, achieving high-resolution imaging of brain structures remains a formidable challenge. Consequently, while the immediate diagnostic value of current GQD technology lies in biofluid sensing, further intensive research is essential to push the boundaries of these materials toward deep-tissue brain imaging.

### 3.5. Safety and Toxicity Profiles and the Therapeutic Window

The greatest barrier to the clinical translation of GQDs is that the therapeutic window has not been measured. Whether the margin between the effective anti-inflammatory dose and the toxic dose is narrow or wide cannot be stated from the present literature, because efficacy and toxicity have been measured in different materials, models, and endpoints.

Reported toxicity mechanisms operate at two levels. At the cellular level, exposure to pristine GQDs induces phenomena including ferroptosis driven by glutathione depletion and lipid peroxidation [122], while N-doped GQDs cause mitochondrial oxidative stress together with disruption of calcium homeostasis [116,117]. In these settings, treated cells exhibit cytosolic iron overload alongside downregulated GPX4 and SLC7A11, directly fueling lipid peroxidation as detected by C11-Bodipy, MDA, and 4-HNE assays. Because Fer-1 pretreatment effectively prevents this cytotoxicity, these findings establish a reliable assay panel to identify key therapeutic targets for counteracting GQD-mediated ferroptosis. In addition, apoptosis and inflammatory responses via the p38 MAPK pathway [123] and, under blue-light irradiation, autophagic cell death [115] have been reported. At the organismal level, developmental toxicity and neurobehavioral changes linked to surface functional groups have been documented. For instance, aminated GQDs (10 nm or smaller) accumulated in zebrafish brain tissue and induced anxiety-like and aggressive behavior that persisted for at least 14 days [124]. In a comparison of four types of GQDs, only the aminated material arrested embryonic development and reduced survival at 100 and 200 μg/mL, and exposure at 100 μg/mL altered expression of genes related to potassium/calcium channels and spliceosome [79].

Surface functional-group identity, concentration, exposure duration, and dopant element are identified as the key determinants of toxicity. In a comparison of three GQDs, hydroxylated material caused significant cell death at 100 μg/mL, whereas carboxylated and aminated materials showed no cytotoxicity across the tested concentration range, confirming surface chemistry as the primary determinant [75]. Furthermore, the carboxylated GQDs administered to mice at 5 and 10 mg/kg over a 21-day period caused no organ damage [16]. Additionally, a material exhibiting less than 10% cytotoxicity even at 250 μg/mL has been reported [118], illustrating how widely toxicity thresholds vary by material. Neurotoxicity assessment frameworks for nanomaterials generally also highlight this material dependence as a core challenge [17]. The dual antioxidant and pro-oxidant behavior of GQDs arises from electronic variations within the same π-system and oxygen-containing functional groups, rather than differences in material type. Specifically, radical scavenging is favored by low defect density and a high abundance of phenolic and carboxylic groups. Conversely, edge and vacancy defects introduce mid-gap states that promote pro-oxidant oxygen reactions. This distinction is further observed in nitrogen-doped GQDs, where graphitic nitrogen facilitates scavenging while pyridinic nitrogen promotes catalytic activity. Although Raman and XPS analyses could effectively organize these reported outcomes, these critical indices have not been systematically reported in the literature. Key reports are summarized in Table 3.

As illustrated in Table 3, efficacy and toxicity are measured in different materials, models, and endpoints, so a safety margin on a common basis cannot be computed. When lung, liver, and kidney pathology after respiratory exposure [93] and dopaminergic neurotoxicity in invertebrate models [120] are also taken into account, it becomes evident that future studies should quantify the therapeutic window numerically by measuring anti-inflammatory efficacy and ferroptosis induction concurrently within the same synthesis batch.

## 4. Discussion

This review organizes the literature around the premise that GQDs can intervene simultaneously in the two pathological axes of neurodegenerative disease, pathogenic protein aggregation and microglia-mediated neuroinflammation. Rather than repeating the individual findings summarized above, the present discussion compares GQDs with existing therapies, considers complementarity among materials, and acknowledges the limitations of the evidence base.

Compared with established antibody therapy, the distinguishing feature of GQDs lies in their site of action. Aβ-targeting antibodies promote microglia-mediated removal of extracellular aggregates, yet their BBB transport efficiency is low, and they have little access to intracellular protein aggregates [65,66]. GQDs, in contrast, cross the barrier by size- and hydrophobicity-driven passive diffusion and reach intracellular and even intranuclear sites, where they can regulate aggregation during the phase-separation stage [100,102]. Because the two approaches act at complementary sites, they may be viewed less as competitors than as candidates for combination; strategies that couple extracellular clearance with intracellular aggregation control remain an untested but promising direction. It must be noted, however, that antibody therapy rests on phase 3 clinical evidence, whereas GQDs remain at the preclinical stage, so the two are not on equal footing in terms of evidence quality [20]. All evidence discussed here is preclinical: it derives from cell-free assays, cell lines, and animal models, and no human data on GQDs in neurodegenerative disease exist.

Lecanemab and donanemab, antibody-based therapies targeting amyloid plaques, have entered the commercialization phase following successful phase 3 trials and FDA approval. In contrast, various nanomedicine platforms, such as carbon dots, graphene oxide, lipid/polymeric nanoparticles, and extracellular vesicles, have not yet received marketing authorization for neurodegenerative diseases. In the case of GQDs, there is currently a lack of quantitative human efficacy and safety data, along with established dosing regimens. Therefore, for GQDs to function as next-generation therapeutics for neurodegenerative diseases, it is essential to transcend simple demonstrations of principle and instead prioritize the establishment of standardized manufacturing processes and the validation of the quantitative indicators presented herein.

Within the material family, graphene oxide and GQDs are better understood as having complementary roles rather than serving as substitutes for one another. Graphene oxide excels at inhibiting the early stages of aggregation through monomer capture on its broad surface, but protein corona formation and accumulation in the body constrain its systemic use [18,88,93]. GQDs enable finer-grained intervention through mechanisms such as tip-capping and fibril penetration that facilitate disassembly [40,95]; furthermore, their small size favors BBB transport and renal clearance, lowering the burden of in vivo accumulation [36,70]. However, it is premature to conclude that GQDs fully resolve the shortcomings of graphene oxide: they remain subject to corona-dependent masking of surface function [76] and can promote aggregation under some conditions [92]. Rather, the difference between the two materials is primarily a matter of degree.

From the standpoint of therapeutic targets, the expansion of the pathophysiological framework from neuronal loss to neuroinflammation and microenvironment control has given new meaning to the immunomodulatory capacity of GQDs [52,55]. Through autophagy activation, they remove pathogenic triggers [78], while ROS scavenging blocks downstream inflammatory signaling [77]. Furthermore, in animal models, the mitigation of pathological microglial states and functional improvement have been observed [106,109]. These observations, however, rest mostly on phenotype-level readouts; the specific transcriptional state transitions that occur at the single-cell level have not yet been resolved [63]. With state-based nomenclature now established, this is a gap that should be filled first.

The duality inherent in these prospects is a problem that must be solved for precision medicine to be realized. Because the redox activity that confers therapeutic benefit and the mechanisms that cause toxicity arise from the same underlying property [116,117], separating efficacy from harm requires sophisticated design of surface functional groups [41,79,100]. This is not merely a matter of making particles smaller; it is a matter of designing materials that respond selectively to target pathological proteins or to specific microglial states.

The limitations of this review should be stated explicitly. First, the evidence cited for therapeutic effects rests mostly on cell-free and animal experiments, including rodent studies, with no human data available. Second, physicochemical reporting across studies is not standardized, so GQDs bearing the same name often differ in their properties, which restricts direct comparison of effect sizes [42]. Third, negative results are rarely published, so publication bias cannot be excluded. The mechanistic synthesis offered here should therefore be read as a working hypothesis assembled from the observations available to date.

Building on the discussion above, we propose the following priorities for future research. First, establish a standardized physicochemical reporting system: without setting lateral-size distribution, zeta potential, oxygen-containing group composition, dopant bonding state, and quantum yield as minimum reporting items, cross-study comparison will remain impossible [42]. Second, verify findings in human-derived models, including microglia and brain organoids generated from patient-derived induced pluripotent stem cells [63]. Third, quantify the therapeutic window by measuring effective and toxic concentrations in parallel within the same batch and expressing the safety margin numerically [79]. Fourth, explore combination strategies, since the aggregate-disassembly function of GQDs and the extracellular-clearance function of antibody therapy act at complementary sites, meriting verification of combined benefit [40,65]. Fifth, track long-term biodistribution and excretion routes quantitatively; longitudinal data obtained by radiolabeling or quantitative mass spectrometry are a prerequisite for regulatory engagement [20]. Table 4 summarizes the current literature on surface functionalization, heteroatom doping, and these characterization protocols. Given the pervasive lack of contamination monitoring in existing reports, rigorous quality control data remains indispensable for reproducible research.

## 5. Conclusions

GQDs combine three traits, namely BBB permeability, tunable surface chemistry, and redox activity, to intervene in both protein aggregation and neuroinflammation. Unlike 2D graphene oxide, GQDs offer precise control over fiber disassembly against α-synuclein, Aβ, tau, and TDP-43, as well as over liquid–liquid phase separation. At the same time, ROS scavenging through heteroatom doping and modulation of autophagy attenuate markers of pathological microglial activation, pointing toward control of the brain’s immune microenvironment. The clinical value of GQDs will therefore be decided less by the discovery of new efficacy than by how carefully their surfaces are engineered and how rigorously the research priorities set out above are met. Once those conditions are satisfied, GQDs could move beyond single-target therapy to become a platform that controls both pathological proteins and the immune microenvironment of the brain.

## Figures and Tables

**Table 1:** Comparison of GQD synthesis methods.

Category	Top-Down	Bottom-Up	Reference
Precursor	Graphite, GO, carbon fibers, CNTs	Citric acid, glucose, amino acids	[29,33,72,73]
Processes	Oxidative cleavage, solvothermal treatment, electrochemical exfoliation, sonication	Carbonization, pyrolysis, stepwise organic synthesis, microwave-assisted synthesis	[29,33,72,73]
Size control	Difficult; broad distribution	Relatively easy	[33,41,71]
Surface chemistry	Oxygen-rich groups; edge composition hard to control	Facile heteroatom doping; designable composition	[33,41,71,72]
Scale-up	Favorable	Difficult; complex process	[29,33,41]
Main limitation	Batch-to-batch variability	Yield and scalability	[33,41,42]

**Table 2:** Aggregation-modulating mechanisms of GQDs against each target. NPC: Niemann-Pick type C.

Target	Material/Surface	Main Mechanism	Validation	Reference
α-Synuclein	Pristine GQD	Fibrillization inhibition, fibril disassembly, reduced cell-to-cell spread	in vitro, *cellular*, *animal*	[40]
α-Synuclein	Functional-group variants	Dimerization inhibition (group-dependent)	in silico	[94]
Aβ	Pristine GQD	Aggregation inhibition, reduced cytotoxicity	in vitro, cellular	[90]
Aβ	GQD-tramiprosate conjugate	Synergistic aggregation inhibition	in vitro, cellular	[98]
Aβ	N-doped GQD	Doping-dependent anti-amyloid activity	in vitro	[99]
Tau	Biomimetic graphene nanoparticles	Blocked cell-to-cell spread	in vitro, cellular	[104]
Tau	D-cysteine GQD in extracellular vesicles	Improved targeting; less aggregation and spread	cellular	[105]
TDP-43	Halogen-doped GQD (GQD-Cl, Cl-GQD-OH)	Nuclear entry; modulated intranuclear phase separation and aggregation	cellular	[100]
TDP-43	Pristine GQD	Reduced stress-granule-mediated cytoplasmic aggregation and fiber formation	cellular, animal	[102]
Insulin, lysozyme (models)	GO quantum dots (oxygen-group tuned)	Group-dependent fibrillation inhibition	in vitro	[74]
hIAPP	Fluorinated GQD	Reduced aggregation and cytotoxicity	in vitro, cellular	[97]
Cholesterol accumulation (NPC)	Pristine GQD	Reduced lysosomal accumulation; functional rescue	cellular, animal	[106]
Huntingtin	-	No direct report	-	-

**Table 3:** Reported efficacy-toxicity cases according to surface chemistry and exposure conditions.

Material	Model/Assay	Condition	Outcome	Reference
N-doped GQD + RGDS	Corneal epithelial cells; dry-eye mice	10 μg/mL	Intracellular antioxidant activity (efficacy)	[77]
N-doped GQD (7.4 nm)	AD rats (Aβ imaging); viability assay	250 μg/mL	<10% cytotoxicity (safe)	[118]
Carboxylated GQD	Mice, 21 days	5 and 10 mg/kg	No organ damage (safe)	[16]
Pristine GQD	EAE rats (Dark Agouti)	10 mg/kg/day i.p.	CNS and lymph node accumulation; reduced inflammation (efficacy)	[109]
Hydroxylated GQD	A549 cells (WST-1, Annexin V/PI)	100 μg/mL	Significant cell death (toxic)	[75]
Carboxylated, aminated GQD	A549 cells (same study)	≤100 μg/mL (tested)	No cytotoxicity	[75]
Aminated GQD	Zebrafish embryos	50–200 μg/mL; effect at ≥100 μg/mL	Arrested development, lower survival and heart rate (toxic)	[79]
Pristine, GO, carboxylated, aminated GQD	Zebrafish embryos; mRNA-seq	100 μg/mL, 7 days	Altered potassium/calcium channel and spliceosome genes	[79]
Aminated GQD (<10 nm)	Zebrafish (behavior)	Not reported; ≥14 days	Anxiety and aggression ≥ 14 days (developmental neurotoxicity)	[124]
N-doped GQD	BV2 microglia; mouse hippocampus	Not reported	Ferroptosis; mitochondrial oxidative stress, calcium dysregulation	[116,117]
Pristine GQD	RAW264.7 (proteomics, lipidomics)	Not reported	Ferroptosis; glutathione depletion, lipid peroxidation	[122]
Pristine GQD	Activated THP-1 macrophages	Not reported; Response reversed at high vs. low dose	Apoptosis, autophagy, inflammation via p38 MAPK	[123]
Pristine GQD + blue light	U251 glioma cells	470 nm, 1 W	ROS, autophagic cell death (photodynamic)	[115]

**Table 4:** Physicochemical characterization of GQDs in primary studies summarized in this review (NR, not reported).

Reference	GQDs	Material Class	Particle Size	Surface Chemistry	Zeta Potential (mV)	Defect Density	Doping	Synthesis Route
Nurunnabi et al. [16]	Carboxylated GQDs	GQDs	3–6 nm (HR-TEM)	COOH	−23.1	NR	NR	Top-down (acid oxidation)
Pan et al. [28]	Blue-luminescent GQDs	GQDs	5–13 nm (TEM)	C=O, COOH, –OH, C–O–C	NR	1.26	NR	Top-down (hydrothermal cutting of GO)
Peng et al. [29]	Blue/green GQDs	GQDs	1–4 nm (TEM)	–OH, C=O, COOH	NR	0.91	NR	Top-down (carbon fiber)
Hassan et al. [31]	aGQDs (edge-enriched)	GQDs	1–8 nm (TEM)	*sp*^2^ C, C–O, C=O, COOH (XPS)	NR	NR (higher D/G vs. GQDs)	NR	Top-down (sonication + KOH activation)
Zhu et al. [34]	N-GQDs	GQDs	5.3 nm (TEM)	–OH, epoxy, COOH, amide	8	NR (defects noted)	Nitrogen	Top-down (hydrothermal cutting of GO)
Kim et al. [40]	Carboxyl-rich GQDs	GQDs	NR	COOH, PEG-biotin	NR	NR	None	Top-down (carbon fiber)
Qu et al. [72]	N-GQDs	GQDs	2.3–7.1 nm (HR-TEM)	COOH, –OH	NR	NR	Nitrogen (urea or ethylenediamine)	Bottom-up (citric acid, hydrothermal)
Rostampour et al. [74]	GOQDs/rGOQDs	GQDs	2–20 nm (TEM)	O-containing (C=O, C–O, O–H)	+10.7 to −41.5	NR	NR	Bottom-up (citric acid)
Xie et al. [75]	aGQDs/cGQDs/hGQDs	GQDs	3.5–5 nm (TEM)	–NH_2_/–COOH/ –OH	NR	NR	NR	Commercial (XFNANO, Nanjing, China)
Wu et al. [77]	N-GQDs (RGDS@NGQDs)	GQDs	3.5 nm (HR-TEM)	O/N, –NH_2_	−30.2	NR	Nitrogen	Bottom-up (hydrothermal)
Hua et al. [78]	GQDs	GQDs	NR	NR	NR	NR	None	Top-down (carbon black)
Valimukhametova et al. [81]	N-GQDs/Nd-NGQDs	GQDs	3.8–4.6 nm (HR-TEM)	N-functional groups	+1.8 (N-GQDs)/+8.3 (Nd-NGQDs)	NR	Nitrogen, Neodymium	Bottom-up (microwave)
Walton-Raaby et al. [82]	GQD7/GQD28 (in silico models)	GQDs	1.5/3.2 nm (model)	Edge-functionalized; N, O, F doping modeled	NR	NR	NR	In silico (ChemDraw 16, PerkinElmerc)
Ghaeidamini et al. [88]	GQDs/GO	GQDs, GO	65 nm (GQD, DLS)	Weak; carboxyl-rich (GO)	−8	NR	NR	Bottom-up (carbonization)
Liu et al. [90]	GQD1-5 variants	GQDs	8 nm (TEM)	COOH, –OH	Negative	NR	NR	Hydrothermal
Oz et al. [91]	GQDs	GQDs	130–3000 nm (flakes)	O-rich (C–OH, C–O, C=O)	−12	NR	NR	Top-down (GO + thermal)
Wu et al. [93]	N-GQDs/A-GQDs	GQDs	3–4 nm (HR-TEM)	N-doped; amino	NR	NR	Nitrogen; amino	Commercial (XFNANO, Nanjing, China)
Yousaf et al. [97]	FGQDs	GQDs	2.4 nm (HR-TEM)	F, COOH, –OH	NR	NR	Fluorine	Bottom-up
Liu et al. [98]	GQD-T (tramiprosate)	GQDs	15–20 nm (AFM)	COOH, tramiprosate	−15.8/−24.2	NR	None (conjugate)	Top-down
Zhang et al. [100]	Cl-GQDs/Cl-GQDs-OH	GQDs	NR	–OH, C–Cl	−2.58/−9.00	NR (G > D; fewer defects in Cl-GQDs-OH)	Chlorine	Bottom-up (halogenated)
ElMorsy et al. [101]	GAQDs	GQDs	10–15 nm (HR-TEM)	Carboxyl-rich	NR	NR	None	Top-down (graphene acid)
Park et al. [102]	Carboxyl-rich GQDs	GQDs	4.7 nm (TEM)	COOH (FT-IR)	NR	NR	None	Top-down (carbon fiber)
Malishev et al. [103]	L-Lys-/D-Lys-C-dots	Carbon dots (comparator)	4 nm (TEM)	Lysine enantiomer surface groups (XPS, FT-IR)	NR	NR	NR	Bottom-up (lysine carbonization)
Zhu et al. [104]	GQDs/Cys-GQDs/EDA-GQDs	GQDs	8.1 nm (TEM)	COOH; Cys; EDA (FT-IR)	−19.9/−1.46/+1.63	NR	None (functionalized)	Top-down
Zhu et al. [105]	D-GQDs (sEV)	GQDs	8.0 nm (TEM)	D-cysteine; sEV lipid	−1.46/−19.9	NR	None (chiral)	Top-down
Kang et al. [106]	Carboxyl-rich GQDs	GQDs	2.25 nm (TEM)	COOH, –OH (FT-IR)	NR	NR	NR	Top-down (carbon fiber)
Tosic et al. [109]	GQDs (graphite/citric)	GQDs	13.5/23.6 nm (AFM)	O-functionalized	−14.0/−26.3	NR	None	Top-down and bottom-up
Tomić et al. [110]	Small/large GQDs	GQDs	23.6/65.6 nm (AFM)	Low-oxygen, *sp*^2^-rich (XPS: C–O 8.4, C=O 5.1 at%)	−9.4/−9.0	NR	None	Top-down (electrochemical)
Volarevic et al. [111]	Large GQDs	GQDs	40 nm (TEM)	NR	NR	NR (D/G bands)	None	Top-down (laser ablation)
Markovic et al. [115]	GQDs	GQDs	56.6 nm (AFM)	Surface carbon free radical (EPR)	NR	NR (EPR)	NR	Top-down (electrochemical)
Wu et al. [116]	N-GQDs/A-GQDs	GQDs	3–4 nm (TEM)	N-doped; amino	−9.9 (N-GQDs)/−25.9 (A-GQDs), DI water	NR	Nitrogen; amino	Commercial (XFNANO, Nanjing, China)
Wu et al. [117]	N-GQDs	GQDs	3 nm (TEM)	N–H, C=O, C–N	−9.9 (DI water)/−12.2 (DMEM)	NR	Nitrogen	Commercial (XFNANO, Nanjing, China)
Bahman et al. [118]	N-GQDs	GQDs	7.4 nm (TEM)	–OH, –COOH, –NH (FT-IR)	NR	0.99	Nitrogen (30.4 at%)	Bottom-up (citric acid + urea)
Gu et al. [120]	A-GQDs	GQDs	2.5–4.5 nm (HR-TEM)	–NH_2_ (aminated) (FT-IR, XPS)	NR	NR	Amino	Bottom-up + amination
Shao et al. [122]	GQDs	GQDs	5 nm (TEM)	O-functional groups (XPS, FT-IR)	NR	NR	Nitrogen (4.72 at%, XPS)	Commercial (First Graphene, Henderson, Australia)
Qin et al. [123]	GQDs	GQDs	1.5–5.5 nm (HR-TEM)	C–O, C=O (FT-IR)	NR	1.005	NR	Top-down (hydrothermal)

## Data Availability

No new data were created or analyzed in this study. Data sharing is not applicable to this article.

## Supplementary Materials

The following supporting information can be downloaded at: https://www.mdpi.com/article/10.3390/nano16181158/s1.

## Abbreviations

The following abbreviations are used in this manuscript:

Abbreviation	Definition
Aβ	amyloid-β
AD	Alzheimer’s disease
ADME	absorption, distribution, metabolism, and excretion
AFM	atomic force microscopy
ALS	amyotrophic lateral sclerosis
ARIA	amyloid-related imaging abnormalities
BBB	blood–brain barrier
CD	circular dichroism
CNS	central nervous system
CNT	carbon nanotube
DAM	disease-associated microglia
DLS	dynamic light scattering
EAE	experimental autoimmune encephalomyelitis
FDA	U.S. Food and Drug Administration
FT-IR	Fourier-transform infrared spectroscopy
GO	graphene oxide
GQD	graphene quantum dot
HD	Huntington’s disease
hIAPP	human islet amyloid polypeptide
HR-TEM	high resolution transmission electron microscopy
IL-1β	interleukin-1β
MAPK	mitogen-activated protein kinase
MRI	magnetic resonance imaging
NF-κB	nuclear factor κB
NLRP3	NLR family pyrin domain-containing 3
NPC	Niemann-Pick type C
PD	Parkinson’s disease
PET	positron emission tomography
PI	propidium iodide
QD	quantum dot
QD-LED	quantum dot light-emitting diode
RGDS	arginine-glycine-aspartate-serine peptide
ROS	reactive oxygen species
SPR	surface plasmon resonance
TDP-43	TAR DNA-binding protein 43
TEM	transmission electron microscopy
WST-1	water-soluble tetrazolium salt-1
XPS	X-ray photoelectron spectroscopy
