# Supplementary material for: Graphene Quantum Dots in Neurodegeneration: Protein Aggregation and the Microglial State as Therapeutic Targets
Source: Nanomaterials (Basel). 2026 Sep 15;16(18):1158. doi: 10.3390/nano16181158 (PMC13610359; doi:10.3390/nano16181158)
Supplement: Supplementary file 1 [file nanomaterials-16-01158-s001.zip › nanomaterials-4549874-supplementary.pdf]

Graphene Quantum Dots in Microglial Modulation and Neurodegeneration

Supplementary Materials: Literature Search Strategy

1. Scope and Purpose

This document describes the structured literature search performed for the narrative review "Graphene Quantum Dots in Microglial Modulation and Neurodegeneration." Consistent with the journal's guidelines for a narrative (non-systematic) review of this article type, a formal systematic-review protocol and PRISMA reporting are not required, and the article is not presented as a systematic, scoping, or mapping review. The search described below was nevertheless performed in a structured, reproducible manner to ensure comprehensive and balanced coverage of the literature.

2. Information Sources

Literature was retrieved from the following bibliographic databases and search engines:

- PubMed / MEDLINE
- Scopus
- Web of Science
- Google Scholar

The search covered the period from 2010, when graphene quantum dots (GQDs) were first introduced, through 25 August 2026, the date of manuscript submission.

In addition, the reference lists of retrieved review articles and primary studies were hand-searched ("snowballing") to identify additional relevant reports, including sources not captured by the database queries.

3. Search Terms and Boolean Search Strings

Search strings combined GQD and related material terms with terms covering microglial function, neuroinflammation, neurodegeneration, protein aggregation, and the blood–brain barrier. An asterisk (*) denotes truncation. The following representative Boolean strings were used (database-specific syntax was adapted as required):

3.1. Material / intervention terms

```
("graphene quantum dot*" OR "GQD*" OR "graphene quantum dots")
```

3.2. Combined topic strings

```
("graphene quantum dot*" OR "GQD*") AND ("microglia*" OR "microglial")

("graphene quantum dot*" OR "GQD*") AND ("neuroinflammation*" OR "neuro-
inflammatory")

("graphene quantum dot*" OR "GQD*") AND ("neurodegeneration*" OR
"neurodegenerative disease*")

("graphene quantum dot*" OR "GQD*") AND ("protein aggregation*" OR
"protein misfolding")

("graphene quantum dot*" OR "GQD*") AND ("blood-brain barrier" OR "BBB")

("graphene quantum dot*" OR "GQD*") AND ("alpha-synuclein" OR "amyloid-
beta" OR "tau")

("graphene quantum dot*" OR "GQD*") AND ("reactive oxygen species" OR
"oxidative stress")

("graphene quantum dot*" OR "GQD*") AND ("autophagy" OR "ferroptosis" OR
"neurotoxicity")

("graphene quantum dot*" OR "GQD*") AND ("bioimaging" OR "fluorescence" OR
"theranostic")
```

Closely related carbon materials (e.g., carbon dots and graphene-oxide quantum dots) were included only where the source explicitly reported relevance to GQD function or was clearly flagged in the text as a related nanomaterial.

4. Inclusion and Exclusion Criteria

4.1. Inclusion criteria

- Original research, and review articles addressing GQD (or explicitly flagged closely related carbon-dot or graphene-oxide quantum dot) physicochemical properties.
- Reports addressing implications for microglial function, neuroinflammation, protein aggregation, neuroimaging, or neurotoxicity.
- Peer-reviewed publications written in English.

4.2. Exclusion criteria

- Editorials, conference abstracts, and correspondence not reporting substantive data or analysis.
- Studies unrelated to GQD effects on the nervous system or to the review topics listed above.
- Non-English-language articles (unless no English equivalent was available and translation was feasible).

5. Screening and Selection Process

The search and screening were conducted by the authors. Titles and abstracts retrieved by the database searches were screened against the inclusion criteria, and full texts of potentially eligible records were retrieved and assessed. Disagreements regarding eligibility were resolved by discussion and consensus. Reference lists of included reviews and primary articles were hand-searched to capture additional relevant sources.

6. Notes on Reproducibility and Reporting

Because this is a narrative review and not a systematic, scoping, or mapping review, article counts, a formal screening log, and a PRISMA flow diagram are not applicable and are not provided. The search described above documents the systematic and structured approach used to assemble the literature basis of the review, consistent with the statement included in the main text.
